# Moving past the usual suspects: a new framework for aural fullness

**DOI:** 10.1016/j.bjorl.2026.101767

**Published:** 2026-01-29

**Authors:** Roseli Saraiva Moreira Bittar, Lucas Resende Lucinda Mangia, Raquel Mezzalira

**Affiliations:** a Faculdade de Medicina da Universidade de São Paulo (FMUSP), Department of Otolaryngology, São Paulo, SP, Brazil; b Universidade Federal do Paraná (UFPR), Department of Otolaryngology, Curitiba, PR, Brazil; c Universidade Estadual de Campinas (UNICAMP), Department of Otolaryngology, Campinas, SP, Brazil

Aural fullness is a frequent yet often misunderstood symptom in clinical practice. Once viewed mainly through the lens of Ménière’s disease or Eustachian tube dysfunction, it is now clear that this complaint can present a much broader origin. Mounting scientific evidence shows that it may arise from diverse auditory, vestibular, metabolic, neuromuscular, and systemic conditions. This more comprehensive perspective challenges longstanding assumptions and underscores the need for a careful, mechanism-driven diagnostic approach. In this editorial, we examine how concepts surrounding aural fullness have evolved and why clinicians must now look far beyond traditional explanations when evaluating this symptom.

Even if some older diagnostic terms sound unfamiliar today, they represented the best interpretations available within the medical literature of their time. Ménière’s disease (MD), for example, was once referred to as a cause of “aural vertigo,” terminology that likely reflected early attempts to conceptualize a disorder frequently accompanied by the sensation of a clogged ear. By the late nineteenth century, it was already recognized that aural fullness (AF) is not exclusive to inner-ear disorders.^1^ Historical reports from 1890 even describe removing the tympanic membrane and malleus in an attempt to relieve a patient’s “aural vertigo.” Advances in modern medicine now allow us to formulate hypotheses and draw conclusions that were previously unattainable.

AF refers to a sensation of a “full ear,” which may be intermittent or persist for hours to weeks, often occurring alongside tinnitus, sound distortion, or disorientation. Although strongly associated with MD, otitis media with effusion, and Eustachian tube dysfunction, the symptom itself is nonspecific and does not establish a diagnosis. Yet, symptom-based diagnostic protocols rely on very limited information and tend to oversimplify clinical reasoning. Otoneurological disorders are complex and frequently share manifestations. Thus, they demand diagnostic thinking that goes beyond protocol-driven decision-making.

It is crucial to recognize that multiple anatomical sites can give rise to this sensation. As an initial step, clinicians must investigate conditions capable of producing acute, rapidly progressive, or fluctuating conductive hearing loss. These are primarily external and middle ear disorders, assessed through comprehensive clinical examination, nasoendoscopy, audiometry, and tympanometry. Once these possibilities are addressed, the differential diagnosis of inner-ear conditions must be carefully pursued.

AF may also reflect systemic disturbances affecting hair-cell metabolism. Brazilian authors have contributed substantially to the understanding of how carbohydrate-metabolic alterations influence cochleovestibular function, affecting labyrinthine performance, cochlear activity, and postural stability.^2^ Hormonal factors likewise modulate neural excitability and auditory processing,^3^ particularly in individuals with a sensitized central nervous system, such as migraineurs. Not surprisingly, middle-aged women with vestibular migraine often report AF. These cases are commonly mistaken for MD, especially when tinnitus or vertigo spells coexist. However, audiometry may remain normal despite abnormal electrocochleography. Most patients respond well to tailored lifestyle adjustments and standard migraine therapies. Dietary habits, metabolic and hormonal fluctuations, and sleep deprivation frequently trigger symptoms that mimic MD.

It is also essential to consider osteomuscular dysfunctions as potential causes of AF. For decades, the role of myofascial contracture and tension in head and neck muscles in triggering tinnitus, headache, and dizziness has been recognized. Muscles commonly involved include the temporalis, cervical muscles, and those associated with the hyoid bone. Historically, this relationship between musculoskeletal dysfunction and otologic symptoms was encapsulated in the so-called Costen triad, which described the coexistence of otalgia, tinnitus, and aural fullness in individuals with temporomandibular joint (TMJ) disorders. A detailed neurophysiological explanation suggests that heightened activity in masticatory and cervical musculature increases trigeminal and upper-cervical afferent discharge to brainstem regions such as the spinal trigeminal nucleus, the dorsal cochlear nucleus, and the parabrachial complex. These structures form key hubs where somatosensory and auditory inputs converge. Abnormal excitatory drive in these pathways may alter central gain, disrupt sensory filtering, and distort the integration of pressure-related cues arising from the middle ear. At the efferent level, trigeminal-motor projections to the tensor tympani and tensor veli palatini may modulate middle-ear impedance and Eustachian tube function, producing subtle mechanical changes perceived as fullness. More recently, discussion has resurfaced regarding the significance of tensor tympani syndrome (TTS), first described in the 1980s.^4^ TTS appears to be associated with anxiety, which lowers reflex thresholds, induces muscle spasm, and produces sensations of muffling within the ears. The condition likely involves the mandibular branch of the trigeminal nerve, providing a direct anatomical and pathophysiological link between masticatory-muscle disorders and the tensor tympani within the middle ear. In a cohort of more than one hundred patients with AF who had been unsuccessfully treated for otitis media or inner ear disorders, the vast majority showed TMJ dysfunction. In most cases, symptoms are resolved or markedly improved after targeted TMJ interventions.^5^

Taken together, these observations demonstrate that aural fullness is not a monolithic entity but rather a shared manifestation of diverse pathophysiological processes. As our understanding continues to expand, it becomes increasingly evident that relying on historical associations or narrow diagnostic frameworks risk overlooking clinically relevant mechanisms and delaying effective treatment. Ultimately, redefining AF as a multifactorial symptom encourages clinicians to move beyond traditional paradigms and toward a more precise, evidence-informed evaluation of the patients who present with this complaint.

## ORCID ID

Roseli Saraiva Moreira Bittar: 0000-0001-8731-8908

Lucas Resende Lucinda Mangia: 0000-0003-3443-3640

Raquel Mezzalira: 0000-0002-0279-8007

## Funding

There is no financial or material support.

## Data availability statement

The authors declare that all data are available in repository.

## Declaration of competing interest

The authors declare no conflicts of interest.
